# DIGNITY: Initial Manual and Tool Development With Community Advisory Board

**DOI:** 10.1093/geroni/igaf122.2068

**Published:** 2025-12-31

**Authors:** Nicole Osevala, Liza Behrens, Susan Ryan, Jennifer Kraschnewski, Kimberly Van Haitsma, Marie Boltz

**Affiliations:** Penn State Health, Hummelstown, Pennsylvania, United States; The Pennsylvania State University, University Park, Pennsylvania, United States; Center for Innovation, Linthicum, Maryland, United States; Penn State College of Medicine, Hershey, Pennsylvania, United States; The Pennsylvania State University, University Park, Pennsylvania, United States; The Pennsylvania State University, University Park, Pennsylvania, United States

## Abstract

Successful translation of scientific discoveries into health improvements requires community involvement in all research stages. Recruiting nursing home (NH) stakeholders has been challenging due to systemic issues highlighted during the COVID-19 pandemic. This project leverages existing NH community stakeholders to co-develop the DIGNITY program for future testing. A community advisory board (CAB) of NH stakeholders was established using purposive sampling. Semi-structured sequential focus groups collected stakeholder opinions on the development, refinement, implementation, and evaluation of the DIGNITY program for rural NH environments. Stakeholders completed a demographic survey and reviewed the drafted DIGNITY program intervention manual. Fourteen NH stakeholders, including academic and clinical experts (n = 5), NH administrators/leaders (n = 3), direct care workers (n = 2), regulators (n = 1), and ombudsman (n = 3), participated in four focus group sessions between October and December 2022. Qualitative content analysis of transcript data indicated that stakeholders found the DIGNITY program relevant and feasible for assisting rural NH staff in honoring residents’ risky preferences for care and activities. Stakeholders identified programmatic areas for modification within six themes: (1) DIGNITY manual formatting, (2) communication, (3) expanding staff roles, (4) addressing residents’ decision-making capacity, (5) potential barriers to implementation, and (6) potential facilitators to implementation. Discussions with stakeholders indicated a need to involve other key stakeholders in rural areas, including residents, family members, direct-care staff, and regulators, in the initial development of DIGNITY. Presented by a participating physician clinician, the discussion will provide a pragmatic participatory lens for improvements made to the DIGNITY program.

